# Lack of HLA predominance and HLA shared epitopes in biliary Atresia

**DOI:** 10.1186/2193-1801-2-42

**Published:** 2013-02-08

**Authors:** Cara L Mack, Kirsten M Anderson, Michael T Aubrey, Philip Rosenthal, Ronald J Sokol, Brian M Freed

**Affiliations:** 1 Departments of Medicine and Immunology, Division of Allergy and Clinical Immunology, University of Colorado School of Medicine, 80045 Aurora, CO USA; 2 Clinimmune Labs, 12635 E Montview Blvd. Suite 300, 80045 Aurora, CO USA; 3 Department of Pediatrics, Division of Pediatric Gastroenterology, Digestive Health Institute, Children’s Hospital Colorado, Hepatology and Nutrition, 13123 East 16th Ave. B290, 80045 Aurora, CO USA; 4 The Liver Center, University of CA San Francisco, 500 Parnassus Ave, 94143 San Francisco, CA USA

## Abstract

Biliary atresia (BA) is characterized by progressive inflammation and fibrosis of bile ducts. A theory of pathogenesis entails autoimmune-mediated injury targeting bile duct epithelia. One of the strongest genetic associations with autoimmunity is with HLA genes. In addition, apparently dissimilar HLA alleles may have similar antigen-binding sites, called shared epitopes, that overlap in their capacity to present antigens. In autoimmune disease, the incidence of the disease may be related to the presence of shared epitopes, not simply the HLA allelic association. Aim: To determine HLA allele frequency (high-resolution genotyping) and shared epitope associations in BA. Results: Analysis of every allele for HLA-A, -B, -C, -DRB1, -DPB1 and -DQB1 in 180 BA and 360 racially-matched controls did not identify any significant HLA association with BA. Furthermore, shared epitope analysis of greater than 10 million possible combinations of peptide sequences was not different between BA and controls. Conclusions: This study encompasses the largest HLA allele frequency analysis for BA in the United States and is the first study to perform shared epitope analysis. When controlling for multiple comparisons, no HLA allele or shared epitope association was identified in BA. Future studies of genetic links to BA that involve alterations of the immune response should include investigations into defects in regulatory T cells and non-HLA linked autoinflammatory diseases.

**Electronic supplementary material:**

The online version of this article (doi:10.1186/2193-1801-2-42) contains supplementary material, which is available to authorized users.

Biliary atresia is the most common neonatal cholestatic disorder, occurring in approximately 1 in 10,000-15,000 live births in the United States. The disease is characterized by complete fibrotic obliteration of the lumen of all or part of the extrahepatic biliary tree within three months of life and progressive inflammation and fibrosis of intrahepatic bile ducts (Sokol et al. 2003). There are two proposed types of BA; the embryonic form and the perinatal form. The embryonic form (~15% of cases) may be due to defective development of the extrahepatic biliary tract and is associated with other congenital anomalies. The perinatal or acquired form occurs in ~85% of cases and the bile duct damage has been theorized to be due to an initial virus infection of the biliary tree that triggers a secondary autoimmune-mediated injury targeting bile duct epithelia. The continued autoimmune response would lead to progressive intrahepatic bile duct injury and sclerosis, resulting in cirrhosis (Mack et al. 2007). Despite clinical improvement following a portoenterostomy procedure at the time of diagnosis, up to 80% of children with BA will eventually require liver transplantation (Sokol et al. 2003).

Compelling evidence for autoimmunity has been gained from mouse studies, where autoreactive T cells targeting bile duct epithelia have been identified. Two groups have demonstrated that autoreactive T cells specific to bile duct epithelia are present in the rotavirus-induced mouse model of BA and are associated with bile duct inflammation and injury (Mack et al. 2006; Shivakumar et al. 2007). In vitro analyses demonstrated significant increases in liver T cells from BA mice that generated IFN-γ in response to either virus or self-bile duct epithelial antigens (Mack et al. 2006). In addition, adoptive transfer of liver T cells from BA mice into immunodeficient recipients led to bile duct-specific inflammation and injury (Mack et al. 2006; Shivakumar et al. 2007). This induction of bile duct pathology occurred in the absence of detectable transferred virus, suggesting that bile duct antigens were the target of the T cells. Human studies pertaining to autoimmunity in BA are limited. Identification of oligoclonal populations, defined as T cells expressing similar T cell receptor variable regions of the β-chain, from BA livers at the time of diagnosis suggests specific antigen-driven inflammation (Mack et al. 2007). It remains unknown what the potential antigens are (e.g. viral proteins, bile duct epithelial proteins) that are responsible for T cell activation and bile duct injury.

One of the strongest genetic associations with autoimmunity is with the human leukocyte antigen (HLA) genes. HLA is a region on chromosome 6 that contains more than 50 genes known to be involved in the immune response. HLA class I (HLA-A,-B and -C) are single polypeptide chains that present endogenous peptides to CD8^+^ T cells and HLA class II molecules (HLA-DR, -DP and -DQ) are heterodimers expressed predominantly on hematopoietic cells that present exogenous peptides to CD4^+^ helper T cells (Abbas && Lichtman 2000). HLA associations with BA have been reported with conflicting results (Silveira et al. 1993; A-Kader et al. 2002; Donaldson et al. 2002; Yuasa et al. 2005), mainly due to the sample size analyzed and the level of resolution used to identify the HLA alleles.

The vast majority of HLA polymorphisms are located within the antigen-binding cleft of the HLA molecule that comes in contact with the peptide or T cell receptor. Peptide binding to HLA-DRB1 molecules, for example, is controlled by 6 pockets within the cleft, each pocket with multiple polymorphic amino acids that create millions of potential peptide binding epitopes (Stern et al. 1994). The peptide fits into a binding groove in the HLA molecule; the residues that are available to interact with peptides in the HLA are located in this groove, and are found to be highly polymorphic among the population, with different residues corresponding to different HLA alleles. Apparently dissimilar HLA alleles may have similar antigen-binding sites, called shared epitopes, and thereby overlap in their capacity to present antigens (Gregersen et al. 1987). Thus in autoimmune and immune-mediated diseases, the incidence and severity of the disease may be related to the presence of shared epitopes, not simply the HLA allelic association (Klareskog et al. 2004).

In this study we sought to determine HLA allele frequency and shared epitope associations in BA patients in the United States, through high-resolution HLA genotyping of all class I and II alleles as well as shared epitope analysis. Identification of potential HLA associations with BA would provide clues to the immune-mediated pathogenesis of this disease.

## Methods

### Study subjects

#### Biliary atresia

Peripheral blood samples were obtained from participants already enrolled in two ongoing NIDDK-funded clinical studies that are being conducted through the Childhood Liver Disease Research and Education Network (ChiLDREN). The NIDDK-funded repository at Rutger’s University processed the blood samples and either stored the DNA directly or developed EBV transformed cell lines, followed by DNA extraction. Our study utilized DNA samples from patients with the perinatal/acquired form of BA, excluding those BA subjects with biliary atresia splenic malformation syndrome and those with other major congenital malformations. Information obtained at the time of sample collection or during the course of the research studies included: sex, race, ethnicity, age at sample collection, age at liver transplant, and outcome (alive with native liver, death or transplant). A total of 178 BA patient DNA samples were available for analysis (Table 1): 76 patients (42.7%) had undergone liver transplant or died within the first two years of life (severe course); 71 patients (39.9%) were 5 years of age or older and had not yet received a liver transplant (mild course); and 31 (17.4%) were between the ages of 2-5 years and were with or without liver transplant (moderate course).

**Table 1:** **Racial distribution of subjects**

Race	BA ***n*** (%)	Controls ***n*** (%)
HLA-A,B		
Black	25 (14.2)	47 (14.3)
Caucasian	114 (64.8)	213 (64.7)
Hispanic	23 (13.1)	43 (13.1)
Asian	14 (8.0)	26 (7.9)
Total	176	329
HLA-C		
Black	25 (14.1)	50 (14.3)
Caucasian	115 (65.0)	226 (64.6)
Hispanic	23 (13.0)	46 (13.1)
Asian	14 (7.9)	28 (8.0)
Total	177	350
HLA-DP		
Caucasian	111 (100)	91 (100)
HLA-DQ		
Black	25 (14.0)	48 (14.0)
Caucasian	116 (65.2)	223 (65.2)
Hispanic	23 (12.9)	44 (12.9)
Asian	14 (7.9)	27 (7.9)
Total	178	342
HLA-DR		
Black	25 (14.0)	96 (14.0)
Caucasian	116 (65.2)	446 (65.2)
Hispanic	23 (12.9)	88 (12.9)
Asian	14 (7.9)	54 (7.9)
Total	178	684

#### Controls

Access to complete HLA genotyping on over 6,600 cord blood samples from the state of Colorado was available through ClinImmune Labs. Two control samples were analyzed for every one BA sample (n=329-350) with the exceptions of HLA-DP (n=91) and HLA-DR (n=684). There were slight differences in the availability of various racial and ethnic groups in the control samples, therefore, racially and ethnically-balanced groups for each of the various HLA molecules were created from sequentially-selected controls subjects (Table 1).

### High resolution HLA genotyping

High resolution allele typing was performed for HLA-A, HLA-B, HLA-C, HLA-DRB1, HLA-DQB1, and HLA-DPB1. Automated capillary electrophoresis sequencing (sequenced-based typing, SBT) was performed on all samples with fluorescent dye technologies (ABI Big Dye, version 1.1). A generic PCR amplification precedes sequencing in both directions using intronic class I primers. HLA-A,B,C sequencing covered exons 2, 3 and 4. Class II SBT for HLA-DRB1, DQB1, and DPB1 covered exon 2. Sequences were interpreted with computer-assisted Assign Software (version 3.5) against current IMGT sequencing libraries (January 2008 to April 2010). The raw sequence electropherograms were archived for future retrieval. Every homozygous sequencing result was confirmed with a secondary technology, such as Luminex SSO (One Lambda) or PCR SSP (Life Technologies).

### Sample size calculation for HLA genotyping

Power calculations, based on Chi square test with continuity correction, without correction for multiple comparisons, was used to determine the sample size required to find a statistically significant difference between a control group frequency of an HLA allele and the BA frequency with a two-sided test using power of 0.8 and a significance level of 0.05. In order to detect a 10% increased frequency of a given HLA allele in BA versus control (i.e. 35% frequency in BA versus 25% in controls), a minimum of 157 patient samples was required.

### Statistical analysis of HLA allele frequency

The number of individuals carrying at least one copy of the allele were counted and compared with the number of individuals not carrying the allele for BA patients and controls, and similarly for comparisons between the mild and severe course. The significance for each allele was calculated using either a Chi square test or Fisher's exact test, as appropriate depending on the number of subjects in each contingency table. The resulting *P* value for each allele (*Pu*= uncorrected *P* value) was corrected for multiple hypotheses testing using the false discovery rate (FDR) method of Benjamini and Yekutieli (2005), to control the expected number of Type I errors (*Pc*= corrected *P* value). This method did not assume independence of tests as required by some FDR methods, and provided more power than the conservative Bonferroni correction.

### Epitope analysis

Epitope analysis was performed using the R software package version 2.6.1 (available online at http://www.r-project.org). Combinations of 1-5 polymorphic amino acids at positions 8-93 of HLA molecules DRB1, DPB1 and DQB1, as well as combinations of up to 4 polymorphic residues at positions 2-182 of HLA molecules A, B and C were considered possible epitopes. Polymorphic residues outside of these ranges are unlikely to influence peptide binding or T cell receptor interactions, and were not considered in this analysis. The number of individuals carrying at least one copy of the possible epitope was compared with the number of individuals not carrying it in both the BA group and controls, and similarly for comparisons between the mild and severe course. The epitope distribution among the patient and control populations was calculated by 2 &× 2 contingency tables and analyzed with either Fisher&’s exact test or Pearson&’s chi-square test as appropriate. The *P* value for each epitope was corrected for multiple comparisons using the false discovery rate method described by Benjamini and Yekutieli (2005) to control for Type I statistical errors.

## Results

### HLA alleles in BA

Allele analysis for HLA-A, -B, -C, -DRB1, -DPB1 and -DQB1 in BA and racially-matched healthy controls did not identify any significant HLA association with BA (Additional file 1: Table S1). Analysis of each allele independently identified two possible alleles that may have been associated with BA: HLA-A*30:02 and -DRB1*15:01. However, neither was statistically significant when controlled for multiple comparisons. Secondly, we sought to determine if there was evidence for a HLA predominance in BA based on the severity of the disease: severe disease course (death or liver transplant in the first 2 years of life) vs. mild disease course (greater than 5 years of age and alive with their native liver). A comparison of severe and mild BA versus the controls yielded no significant association. Additionally, there were no significant differences between severe and mild BA when compared to each other (Additional file 2: Table S2).

### HLA shared epitope analysis in BA

An epitope analysis was performed using all possible combinations of 1-4 amino acids for HLA-A (2,326,697 epitopes), HLA-B (3,129,345 epitopes), HLA-C (685,369 epitopes), and 1-5 amino acids for HLA-DRB1 (2,370,369 epitopes), HLA-DQB1 (1,524,709 epitopes) and HLA-DPB1 (65,853 epitopes). Shown in Tables 2, 3, 4, 5, 6, 7 are the data for single amino acid combinations for each HLA group. None of 10,102,332 possible HLA epitopes was significantly different between BA patients and controls. Shared epitope analysis was also performed comparing the severe and mild disease courses of BA. Single amino acid epitope analysis on polymorphic amino acid residues 2-182 for HLA-A, -B, -C and residues 8-93 for HLA-DRB1, -DPB1, and -DQB1 was performed in these 2 groups. No significant differences were identified between the severe and mild BA patients compared with controls. Finally, the severe and mild BA groups were compared to each other. 580 single amino acid epitopes across HLA-A, -B, -C, -DRB1, -DQB1, and -DPB1were compared between the severe and mild forms of BA and no significant differences between the two groups were identified (data not shown).

**Table 2:** HLA-A single amino acid polymorphisms**

Positions^†^	Amino acid	Patients (176) n (%)	Controls (329) n (%)	Odds Ratio	***Pu*** value*	***Pc*** value*
9	F	134(76.14)	251(76.29)	1.0	0.97	NS
9	S	47(26.70)	108(32.83)	0.8	0.16	NS
9	T	31(17.61)	55(16.72)	0.9	0.80	NS
9	Y	70(39.77)	123(37.39)	0.9	0.60	NS
44	K	44(25.00)	84(25.53)	1.0	0.90	NS
56	R	21(11.93)	39(11.85)	1.0	0.98	NS
62	E	33(18.75)	89(27.05)	0.7	0.04	NS
62	G	82(46.59)	160(48.63)	1.0	0.66	NS
62	Q	113(64.20)	197(59.88)	0.9	0.34	NS
62	R	57(32.39)	94(28.57)	0.9	0.37	NS
63	N	57(32.39)	94(28.57)	0.9	0.37	NS
65	G	32(18.18)	89(27.05)	0.7	0.03	NS
66	K	104(59.09)	223(67.78)	0.9	0.05	NS
66	N	152(86.36)	257(78.12)	0.9	0.02	NS
67	M	44(25.00)	84(25.53)	1.0	0.90	NS
70	H	157(89.20)	310(94.22)	0.9	0.04	NS
70	Q	92(52.27)	164(49.85)	1.0	0.60	NS
73	I	20(11.36)	37(11.25)	1.0	0.97	NS
74	D	163(92.61)	296(89.97)	1.0	0.33	NS
74	H	82(46.59)	160(48.63)	1.0	0.66	NS
76	A	66(37.50)	112(34.04)	0.9	0.44	NS
76	E	55(31.25)	119(36.17)	0.9	0.27	NS
76	V	151(85.80)	270(82.07)	1.0	0.28	NS
77	D	151(85.80)	270(82.07)	1.0	0.28	NS
77	N	102(57.95)	187(56.84)	1.0	0.81	NS
79	R	46(26.14)	111(33.74)	0.8	0.08	NS
80	I	46(26.14)	111(33.74)	0.8	0.08	NS
81	A	46(26.14)	111(33.74)	0.8	0.08	NS
82	L	46(26.14)	111(33.74)	0.8	0.08	NS
83	R	46(26.14)	111(33.74)	0.8	0.08	NS
90	D	85(48.30)	150(45.59)	0.9	0.56	NS
95	I	152(86.36)	259(78.72)	0.9	0.04	NS
95	L	37(21.02)	104(31.61)	0.7	0.01	NS
95	V	76(43.18)	145(44.07)	1.0	0.85	NS
97	I	109(61.93)	184(55.93)	0.9	0.19	NS
97	M	74(42.05)	175(53.19)	0.8	0.02	NS
97	R	99(56.25)	198(60.18)	0.9	0.39	NS
99	F	32(18.18)	89(27.05)	0.7	0.03	NS
105	P	92(52.27)	167(50.76)	1.0	0.75	NS
105	S	158(89.77)	297(90.27)	1.0	0.86	NS
107	G	164(93.18)	296(89.97)	1.0	0.23	NS
107	W	82(46.59)	161(48.94)	1.0	0.62	NS
114	H	108(61.36)	232(70.52)	0.9	0.04	NS
114	Q	48(27.27)	85(25.84)	0.9	0.73	NS
114	R	109(61.93)	193(58.66)	0.9	0.48	NS
116	D	142(80.68)	241(73.25)	0.9	0.06	NS
116	Y	108(61.36)	232(70.52)	0.9	0.04	NS
127	K	115(65.34)	250(75.99)	0.9	0.01	NS
127	N	143(81.25)	243(73.86)	0.9	0.06	NS
142	I	157(89.20)	284(86.32)	1.0	0.35	NS
142	T	94(53.41)	191(58.05)	0.9	0.32	NS
144	Q	77(43.75)	138(41.95)	1.0	0.70	NS
145	H	94(53.41)	191(58.05)	0.9	0.32	NS
145	R	157(89.20)	284(86.32)	1.0	0.35	NS
149	T	28(15.91)	41(12.46)	0.8	0.28	NS
150	V	44(25.00)	84(25.53)	1.0	0.90	NS
151	R	59(33.52)	112(34.04)	1.0	0.91	NS
152	A	57(32.39)	119(36.17)	0.9	0.40	NS
152	E	65(36.93)	96(29.18)	0.8	0.07	NS
152	V	140(79.55)	291(88.45)	0.9	0.01	NS
156	L	143(81.25)	257(78.12)	1.0	0.41	NS
156	Q	40(22.73)	101(30.70)	0.7	0.06	NS
156	R	44(25.00)	84(25.53)	1.0	0.90	NS
156	W	49(27.84)	93(28.27)	1.0	0.92	NS
158	V	44(25.00)	84(25.53)	1.0	0.90	NS
161	D	44(25.00)	56(17.02)	0.7	0.03	NS
163	R	79(44.89)	144(43.77)	1.0	0.81	NS
166	D	73(41.48)	155(47.11)	0.9	0.23	NS
166	E	163(92.61)	295(89.67)	1.0	0.28	NS
167	G	73(41.48)	155(47.11)	0.9	0.23	NS
167	W	163(92.61)	295(89.67)	1.0	0.28	NS

*Pu, uncorrected *P* values; *Pc*, corrected *P* values.

† Polymorphic amino acid positions 3, 9, 12, 14, 17, 19, 31, 35, 43, 44, 56, 62, 63, 65, 66, 67, 70, 73, 74, 76, 77, 79, 80, 81, 82, 83, 90, 95, 97, 99, 102, 105, 107, 109, 114, 116, 127, 142, 144, 149, 150, 151, 152, 156, 158, 161, 163, 166, 167, 171.

** Not displaying amino acid residues that were present in 90% or greater in both patient and control groups, or were present in 10% or fewer in both patient and control groups.

**Table 3:** HLA-B single amino acid polymorphisms**

Positions^†^	Amino acid	Patients (176) n (%)	Controls (329) n (%)	Odds Ratio	***Pu*** value*	***Pc*** value*
9	D	29(16.48)	52(15.81)	1.0	0.84	NS
9	H	57(32.39)	118(35.87)	0.9	0.43	NS
11	A	154(87.50)	303(92.10)	1.0	0.09	NS
11	S	85(48.30)	174(52.89)	0.9	0.33	NS
12	M	149(84.66)	298(90.58)	0.9	0.05	NS
12	V	95(53.98)	187(56.84)	0.9	0.54	NS
24	A	92(52.27)	172(52.28)	1.0	1.00	NS
24	S	110(62.50)	205(62.31)	1.0	0.97	NS
24	T	88(50.00)	173(52.58)	1.0	0.58	NS
32	L	79(44.89)	162(49.24)	0.9	0.35	NS
41	T	80(45.45)	157(47.72)	1.0	0.63	NS
45	E	115(65.34)	210(63.83)	1.0	0.74	NS
45	K	72(40.91)	147(44.68)	0.9	0.42	NS
45	M	43(24.43)	83(25.23)	1.0	0.84	NS
45	T	71(40.34)	137(41.64)	1.0	0.78	NS
46	A	43(24.43)	83(25.23)	1.0	0.84	NS
62	G	19(10.80)	37(11.25)	1.0	0.88	NS
63	E	122(69.32)	245(74.47)	0.9	0.22	NS
63	N	136(77.27)	269(81.76)	0.9	0.23	NS
65	R	22(12.50)	40(12.16)	1.0	0.91	NS
66	N	22(12.50)	40(12.16)	1.0	0.91	NS
67	C	49(27.84)	78(23.71)	1.2	0.31	NS
67	F	68(38.64)	139(42.25)	0.9	0.43	NS
67	M	22(12.50)	40(12.16)	1.0	0.91	NS
67	S	110(62.50)	215(65.35)	1.0	0.52	NS
67	Y	56(31.82)	97(29.48)	1.1	0.59	NS
69	A	75(42.61)	144(43.77)	1.0	0.80	NS
70	Q	55(31.25)	96(29.18)	1.1	0.63	NS
70	S	22(12.50)	40(12.16)	1.0	0.91	NS
71	A	78(44.32)	146(44.38)	1.0	0.99	NS
74	D	107(60.80)	193(58.66)	1.0	0.64	NS
74	Y	151(85.80)	291(88.45)	1.0	0.39	NS
77	N	94(53.41)	183(55.62)	1.0	0.63	NS
77	S	155(88.07)	276(83.89)	1.0	0.21	NS
80	I	50(28.41)	106(32.22)	0.9	0.38	NS
80	N	154(87.50)	277(84.19)	1.0	0.32	NS
80	T	66(37.50)	118(35.87)	1.0	0.72	NS
81	A	94(53.41)	183(55.62)	1.0	0.63	NS
81	L	158(89.77)	290(88.15)	1.0	0.58	NS
82	L	104(59.09)	197(59.88)	1.0	0.86	NS
82	R	154(87.50)	277(84.19)	1.0	0.32	NS
83	G	154(87.50)	277(84.19)	1.0	0.32	NS
83	R	104(59.09)	197(59.88)	1.0	0.86	NS
94	I	85(48.30)	163(49.54)	1.0	0.79	NS
95	I	91(51.70)	166(50.46)	1.0	0.79	NS
95	L	143(81.25)	256(77.81)	1.0	0.37	NS
95	W	49(27.84)	106(32.22)	0.9	0.31	NS
97	R	139(78.98)	245(74.47)	1.1	0.26	NS
97	S	80(45.45)	145(44.07)	1.0	0.77	NS
97	T	35(19.89)	83(25.23)	0.8	0.18	NS
97	W	24(13.64)	23(6.99)	2.0	0.01	NS
103	L	70(39.77)	103(31.31)	1.3	0.06	NS
113	Y	85(48.30)	147(44.68)	1.1	0.44	NS
114	D	133(75.57)	241(73.25)	1.0	0.57	NS
114	N	122(69.32)	227(69.00)	1.0	0.94	NS
116	D	52(29.55)	103(31.31)	0.9	0.68	NS
116	F	47(26.70)	67(20.36)	1.3	0.10	NS
116	L	28(15.91)	48(14.59)	1.1	0.69	NS
116	S	72(40.91)	135(41.03)	1.0	0.98	NS
116	Y	108(61.36)	221(67.17)	0.9	0.19	NS
131	R	89(50.57)	173(52.58)	1.0	0.67	NS
131	S	158(89.77)	299(90.88)	1.0	0.69	NS
143	S	14(7.95)	37(11.25)	0.7	0.24	NS
147	L	14(7.95)	37(11.25)	0.7	0.24	NS
152	E	108(61.36)	188(57.14)	1.1	0.36	NS
152	V	155(88.07)	281(85.41)	1.0	0.41	NS
156	D	63(35.80)	111(33.74)	1.1	0.64	NS
156	L	148(84.09)	275(83.59)	1.0	0.88	NS
156	R	40(22.73)	80(24.32)	0.9	0.69	NS
156	W	24(13.64)	37(11.25)	1.2	0.43	NS
158	T	17(9.66)	33(10.03)	1.0	0.89	NS
163	E	71(40.34)	149(45.29)	0.9	0.29	NS
163	L	123(69.89)	248(75.38)	0.9	0.18	NS
163	T	87(49.43)	148(44.98)	1.1	0.34	NS
167	S	46(26.14)	87(26.44)	1.0	0.94	NS
171	H	46(26.14)	93(28.27)	0.9	0.61	NS
177	D	81(46.02)	162(49.24)	0.9	0.49	NS
178	K	50(28.41)	109(33.13)	0.9	0.28	NS
180	E	81(46.02)	162(49.24)	0.9	0.49	NS

*Pu, uncorrected *P* values; *Pc*, corrected *P* values.

† Polymorphic amino acid positions 3, 9, 12, 14, 17, 19, 31, 35, 43, 44, 56, 62, 63, 65, 66, 67, 70, 73, 74, 76, 77, 79, 80, 81, 82, 83, 90, 95, 97, 99, 102, 105, 107, 109, 114, 116, 127, 142, 144, 149, 150, 151, 152, 156, 158, 161, 163, 166, 167, 171.

** Not displaying amino acid residues that were present in 90% or greater in both patient and

control groups, or were present in 10% or fewer in both patient and control groups.

**Table 4:** **HLA-C single amino acid polymorphisms**

Position**	Amino acid	BA (177) ***n*** (%)	Controls (350) ***n*** (%)	Odds Ratio	***Pu*** value^#^	***Pc*** value^#^
9	S	43(24.3)	109(31.1)	0.78	0.12	NS
9	D	106(59.9)	190(54.3)	1.10	0.17	NS
9	Y	125(70.6)	236(67.4)	1.05	0.35	NS
11	S	56(31.6)	132(37.7)	0.84	0.20	NS
14	W	39(22.0)	98(28.0)	0.79	0.16	NS
21	H	61(34.5)	117(33.4)	1.03	0.74	NS
24	S	116(65.5)	208(59.4)	1.10	0.13	NS
24	A	149(84.2)	290(82.9)	1.02	0.50	NS
35	Q	49(27.7)	92(26.3)	1.05	0.68	NS
49	E	39(22.0)	98(28.0)	0.79	0.16	NS
66	N	48(27.1)	96(27.4)	0.99	1.00	NS
73	T	113(63.8)	230(65.7)	0.97	0.80	NS
73	A	136(76.8)	277(79.1)	0.97	0.71	NS
77	N	106(59.9)	227(64.9)	0.92	0.34	NS
77	S	153(86.4)	294(84.0)	1.03	0.30	NS
80	K	106(59.9)	227(64.9)	0.92	0.34	NS
80	N	153(86.4)	294(84.0)	1.03	0.30	NS
90	D	125(70.6)	255(72.9)	0.97	0.73	NS
90	A	129(72.9)	257(73.4)	0.99	0.94	NS
91	R	20(11.3)	31(8.9)	1.28	0.35	NS

*# Pu*, uncorrected *P* values; *Pc*, corrected *P* values.

**Polymorphic amino acid positions analyzed: 6, 9, 11, 14, 16, 21, 24, 35, 45, 49, 52, 63, 66, 69, 73, 76, 77, 80, 90, 91, 94, 95, 97, 99, 103, 113, 114, 116, 138, 143, 147, 152, 156, 163, 170, 173, 175, 177, 178, 180.

Not shown are amino acid residues that were present in 90% or greater in both patient and control groups, or were present in 10% or fewer in both patient and control groups.

**Table 5:** HLA-DR single amino acid polymorphisms**

Positions^†^	Amino acid	Patients (178) n (%)	Controls (684) n (%)	Odds ratio	***Pu*** value*	***Pc*** value*
9	E	103(57.87)	207(30.26)	1.9	0.56	NS
9	K	150(84.27)	291(42.54)	2.0	0.81	NS
10	E	118(66.29)	242(35.38)	1.9	0.29	NS
10	Q	135(75.84)	277(40.50)	1.9	0.17	NS
11	D	32(17.98)	60(8.77)	2.0	0.90	NS
11	G	42(23.60)	77(11.26)	2.1	0.78	NS
11	L	49(27.53)	101(14.77)	1.9	0.63	NS
11	P	54(30.34)	97(14.18)	2.1	0.64	NS
11	S	118(66.29)	242(35.38)	1.9	0.29	NS
12	K	118(66.29)	242(35.38)	1.9	0.29	NS
12	T	138(77.53)	277(40.50)	1.9	0.35	NS
13	F	25(14.04)	53(7.75)	1.8	0.66	NS
13	G	42(23.60)	77(11.26)	2.1	0.78	NS
13	H	42(23.60)	76(11.11)	2.1	0.72	NS
13	R	49(27.53)	101(14.77)	1.9	0.63	NS
13	S	50(28.09)	96(14.04)	2.0	1.00	NS
13	Y	103(57.87)	211(30.85)	1.9	0.40	NS
14	E	42(23.60)	77(11.26)	2.1	0.78	NS
14	K	176(98.88)	338(49.42)	2.0	1.00	NS
16	H	25(14.04)	53(7.75)	1.8	0.66	NS
16	Q	176(98.88)	339(49.56)	2.0	1.00	NS
25	Q	42(23.60)	77(11.26)	2.1	0.78	NS
25	R	176(98.88)	338(49.42)	2.0	1.00	NS
26	F	39(21.91)	76(11.11)	2.0	0.94	NS
26	L	45(25.28)	73(10.67)	2.4	0.31	NS
26	Y	168(94.38)	322(47.08)	2.0	0.92	NS
28	D	82(46.07)	151(22.08)	2.1	0.68	NS
28	E	163(91.57)	315(46.05)	2.0	0.83	NS
30	C	18(10.11)	23(3.36)	3.0	0.17	NS
30	G	32(17.98)	60(8.77)	2.0	0.90	NS
30	H	42(23.60)	77(11.26)	2.1	0.78	NS
30	L	159(89.33)	316(46.20)	1.9	0.24	NS
31	F	39(21.91)	74(10.82)	2.0	0.94	NS
31	I	177(99.44)	339(49.56)	2.0	1.00	NS
32	H	85(47.75)	165(24.12)	2.0	0.92	NS
32	Y	158(88.76)	318(46.49)	1.9	0.10	NS
33	H	50(28.09)	96(14.04)	2.0	1.00	NS
33	N	171(96.07)	330(48.25)	2.0	0.81	NS
37	L	51(28.65)	95(13.89)	2.1	0.83	NS
37	N	75(42.13)	150(21.93)	1.9	0.71	NS
37	S	78(43.82)	147(21.49)	2.0	0.85	NS
37	Y	96(53.93)	185(27.05)	2.0	0.97	NS
38	L	178(100.00)	342(50.00)	2.0	1.00	NS
40	F	178(100.00)	342(50.00)	2.0	1.00	NS
47	F	123(69.10)	243(35.53)	1.9	0.64	NS
47	Y	134(75.28)	273(39.91)	1.9	0.23	NS
57	D	20(11.24)	44(6.43)	1.7	0.59	NS
57	S	58(32.58)	100(14.62)	2.2	0.43	NS
57	V	169(94.94)	322(47.08)	2.0	0.84	NS
58	A	32(17.98)	63(9.21)	2.0	0.90	NS
58	E	175(98.31)	340(49.71)	2.0	0.34	NS
60	S	58(32.58)	100(14.62)	2.2	0.43	NS
60	Y	175(98.31)	333(48.68)	2.0	0.76	NS
67	F	52(29.21)	107(15.64)	1.9	0.63	NS
67	I	114(64.04)	215(31.43)	2.0	0.79	NS
67	L	123(69.10)	223(32.60)	2.1	0.37	NS
70	D	20(11.24)	35(5.12)	2.2	0.72	NS
70	Q	125(70.22)	228(33.33)	2.1	0.41	NS
70	R	130(73.03)	254(37.13)	2.0	0.76	NS
71	A	43(24.16)	89(13.01)	1.9	0.64	NS
71	E	47(26.40)	78(11.40)	2.3	0.36	NS
71	K	58(32.58)	120(17.54)	1.9	0.57	NS
71	R	139(78.09)	264(38.60)	2.0	0.82	NS
73	A	77(43.26)	134(19.59)	2.2	0.37	NS
73	G	170(95.51)	321(46.93)	2.0	0.55	NS
74	E	26(14.61)	48(7.02)	2.1	0.86	NS
74	L	38(21.35)	70(10.23)	2.1	0.81	NS
74	Q	42(23.60)	77(11.26)	2.1	0.78	NS
74	R	153(85.96)	299(43.71)	2.0	0.64	NS
77	N	38(21.35)	71(10.38)	2.1	0.88	NS
77	T	175(98.31)	338(49.42)	2.0	0.70	NS
78	V	49(27.53)	90(13.16)	2.1	0.77	NS
78	Y	176(98.88)	336(49.12)	2.0	0.72	NS
85	V	178(100.00)	341(49.85)	2.0	1.00	NS
86	G	132(74.16)	265(38.74)	1.9	0.40	NS
86	V	133(74.72)	240(35.09)	2.1	0.27	NS

*Pu, uncorrected *P* values; *Pc*, corrected *P* values.

† Polymorphic amino acid positions 9, 10, 11, 12, 13, 14, 16, 25, 26, 28, 30, 31, 32, 33, 37, 38, 40, 47, 57, 58, 60, 67, 70, 71, 73, 74, 77, 78, 85, 86.

** Not displaying amino acid residues that were present in 90% or greater in both patient and control groups, or were present in 10% or fewer in both patient and control groups.

**Table 6:** HLA-DP single amino acid polymorphisms**

Positions^†^	Amino acid	Patients (111) n (%)	Controls (91) n (%)	Odds Ratio	***Pu*** value*	***Pc*** value*
8	V	59(53.15)	39(42.86)	1.2	0.15	NS
9	H	8(7.21)	11(12.09)	0.6	0.33	NS
9	Y	53(47.75)	31(34.07)	1.4	0.05	NS
11	L	43(38.74)	35(38.46)	1.0	0.97	NS
35	Y	27(24.32)	10(10.99)	2.2	0.01	NS
36	A	85(76.58)	67(73.63)	1.0	0.63	NS
36	V	78(70.27)	66(72.53)	1.0	0.72	NS
55	A	86(77.48)	68(74.73)	1.0	0.65	NS
55	D	75(67.57)	64(70.33)	1.0	0.67	NS
56	A	88(79.28)	71(78.02)	1.0	0.83	NS
56	E	75(67.57)	64(70.33)	1.0	0.67	NS
57	D	36(32.43)	27(29.67)	1.1	0.67	NS
65	L	41(36.94)	26(28.57)	1.3	0.21	NS
69	E	44(39.64)	34(37.36)	1.1	0.74	NS
76	V	45(40.54)	32(35.16)	1.2	0.43	NS
84	D	60(54.05)	40(43.96)	1.2	0.15	NS
84	G	97(87.39)	80(87.91)	1.0	0.91	NS
85	E	60(54.05)	40(43.96)	1.2	0.15	NS
85	G	99(89.19)	80(87.91)	1.0	0.78	NS
86	A	60(54.05)	40(43.96)	1.2	0.15	NS
86	P	99(89.19)	80(87.91)	1.0	0.78	NS
87	M	99(89.19)	80(87.91)	1.0	0.78	NS
87	V	60(54.05)	40(43.96)	1.2	0.15	NS

*Pu, uncorrected *P* values; *Pc*, corrected *P* values.

† Polymorphic amino acid positions 8, 9, 11, 33, 35, 36, 55, 56, 57, 65, 69, 76, 84, 85, 86, 87 ** Not displaying amino acid residues that were present in 90% or greater in both patient and control groups, or were present in 10% or fewer in both patient and control groups.

**Table 7:** HLA-DQ single amino acid polymorphisms**

Positions^†^	Amino acid	Patients (178) n (%)	Controls (342) n (%)	Odds ratio	***Pu*** value*	***Pc*** value*
9	F	56(31.46)	97(28.36)	1.1	0.46	NS
13	A	65(36.52)	133(38.89)	0.9	0.60	NS
14	L	57(32.02)	123(35.96)	0.9	0.37	NS
26	G	69(38.76)	149(43.57)	0.9	0.29	NS
26	L	149(83.71)	266(77.78)	1.1	0.11	NS
26	Y	65(36.52)	133(38.89)	0.9	0.60	NS
28	S	66(37.08)	129(37.72)	1.0	0.89	NS
30	H	84(47.19)	146(42.69)	1.1	0.33	NS
30	S	66(37.08)	129(37.72)	1.0	0.89	NS
30	Y	132(74.16)	265(77.49)	1.0	0.40	NS
37	I	66(37.08)	129(37.72)	1.0	0.89	NS
38	A	147(82.58)	284(83.04)	1.0	0.90	NS
38	V	110(61.80)	237(69.30)	0.9	0.09	NS
45	E	60(33.71)	120(35.09)	1.0	0.75	NS
46	E	66(37.08)	129(37.72)	1.0	0.89	NS
47	F	66(37.08)	129(37.72)	1.0	0.89	NS
52	L	66(37.08)	129(37.72)	1.0	0.89	NS
53	L	148(83.15)	277(80.99)	1.0	0.55	NS
53	Q	122(68.54)	213(62.28)	1.1	0.16	NS
55	L	66(37.08)	129(37.72)	1.0	0.89	NS
55	P	94(52.81)	192(56.14)	0.9	0.47	NS
55	R	130(73.03)	231(67.54)	1.1	0.20	NS
57	A	95(53.37)	179(52.34)	1.0	0.82	NS
57	D	132(74.16)	261(76.32)	1.0	0.59	NS
57	V	54(30.34)	100(29.24)	1.0	0.79	NS
66	D	83(46.63)	167(48.83)	1.0	0.63	NS
67	I	83(46.63)	167(48.83)	1.0	0.63	NS
70	G	110(61.80)	194(56.73)	1.1	0.27	NS
70	R	150(84.27)	280(81.87)	1.0	0.49	NS
71	A	57(32.02)	122(35.67)	0.9	0.41	NS
71	K	66(37.08)	129(37.72)	1.0	0.89	NS
71	T	144(80.90)	277(80.99)	1.0	0.98	NS
74	A	66(37.08)	129(37.72)	1.0	0.89	NS
74	E	144(80.90)	277(80.99)	1.0	0.98	NS
74	S	69(38.76)	149(43.57)	0.9	0.29	NS
75	L	144(80.90)	277(80.99)	1.0	0.98	NS
75	V	116(65.17)	244(71.35)	0.9	0.15	NS
77	R	105(58.99)	225(65.79)	0.9	0.13	NS
77	T	148(83.15)	290(84.80)	1.0	0.62	NS
84	E	122(68.54)	213(62.28)	1.1	0.16	NS
84	Q	148(83.15)	277(80.99)	1.0	0.55	NS
85	L	148(83.15)	277(80.99)	1.0	0.55	NS
85	V	122(68.54)	213(62.28)	1.1	0.16	NS
86	A	114(64.04)	206(60.23)	1.1	0.40	NS
86	E	148(83.15)	277(80.99)	1.0	0.55	NS
87	F	69(38.76)	113(33.04)	1.2	0.19	NS
87	L	148(83.15)	277(80.99)	1.0	0.55	NS
87	Y	69(38.76)	134(39.18)	1.0	0.93	NS
89	G	122(68.54)	213(62.28)	1.1	0.16	NS
89	T	148(83.15)	277(80.99)	1.0	0.55	NS
90	I	122(68.54)	213(62.28)	1.1	0.16	NS
90	T	148(83.15)	277(80.99)	1.0	0.55	NS

*Pu, uncorrected *P* values; *Pc*, corrected *P* values.

† Polymorphic amino acid positions 9, 13, 14, 23, 26, 27, 28, 30, 37, 38, 45, 46,47, 52, 53, 55, 56, 57, 60 66, 67, 70, 71, 74, 75, 77, 84, 85, 86, 87, 89, 90.

** Not displaying amino acid residues that were present in 90% or greater in both patient and control groups, or were present in 10% or fewer in both patient and control groups.

## Discussion

This study encompasses the largest HLA allele frequency analysis for BA in the United States and is the first study to perform shared epitope analysis. When controlling for multiple comparisons, no HLA allele or shared epitope association was identified in BA. In previous HLA association studies, a serological phenotype technique was performed on small numbers of BA patients and identified potential HLA associations with BA HLA-B12 (Silveira et al.^1993^) and HLA-B8 and -DR3(A-Kader et al. 2002). HLA genotyping is now based on DNA-sequencing, which permits a greater number of HLA loci and alleles to be tested with increased accuracy. To that end, Donaldson et al. (Donaldson et al. 2002) genotyped 101 BA children and found no significant differences compared to controls. However, HLA-C was not analyzed and the genotyping was performed at low resolution, which limits the number of alleles that can be analyzed. Our study expands on this work as it measured all class I and class II HLA alleles by high resolution genotyping. A Japanese study of 392 BA patients and 828 controls analyzed 17 HLA-A, 19 HLA-B and 16 HLA-DR antigens (Yuasa et al. 2005). Significantly more BA patients expressed HLA-DR2 (DR15, DR16 in current nomenclature) (39%) compared to controls (30.4%) (*p*_c_=0.03; OR 1.46). Two locus analysis revealed that HLA-DR2 was not independently associated with BA but rather the combined expression of HLA-A24-B52-DR2 was significantly greater in BA patients (14.9%) versus controls (7.36%) (*p*=0.001; OR 2.2), a phenomenon known as linkage disequilibrium. These results suggested that the gene for BA susceptibility is at a locus in *close proximity* to the HLA locus.

One might argue that a larger BA population needed to be studied in order to identify a HLA allele association. Indeed, our power calculations to identify a 5% increase in a specific HLA allele frequency in BA versus controls (i.e. 15% BA versus 10% controls) would have required a sample size of 316 BA patients. However, the shared epitope analysis is extremely sensitive at identifying potential HLA associations and the evaluation of >10 million epitopes most certainly would have identified an association (Freed et al. 2011; Karp et al. 2010). For example, Freed et al. performed shared epitope analysis on patients with rheumatoid arthritis and assessed all possible combinations of up to 5 amino acids within the peptide binding groove of HLA-DRB1 (Freed et al. 2011). Several HLA-DR4 alleles have previously been linked with rheumatoid arthritis, with strong associations with DRB1*04:01, *04:04, and *04:05, and weaker associations with DRB1*01:01, *01:02, *10:01, and *14:02. These disparate alleles had been hypothesized to contribute to rheumatoid arthritis via the presence of a shared epitope at the peptide-binding groove. Of the >2 million epitopes examined, LA^67,74^ (leucine at position 67 and alanine at position 74) exhibited the highest correlation with rheumatoid arthritis susceptibility (*P*=2 x 10^-20^). This same group is presently analyzing for shared epitopes in diabetes and chronic hepatitis C infection in order to predict disease associations and prognoses.

One limitation of our study is that we did not analyze the HLA-DRB3, DRB4, DRB5, DQA1, or DPA1 alleles. We chose not to analyze these few alleles because the literature examining disease associations with these loci is sparse and these loci are linked to other loci that were tested for and therefore would have identified the association. For example, DQA1 is tightly linked to DQB1, so if there were a DQA1 link we would have seen it in the DQB1 analysis. This is similarly true for DRB3, DRB4 and DRB5, albeit they are less tightly linked (Thorsby && Lie 2005; Gough && Simmonds 2007; Shiina et al. 2004).

Based on our findings showing no HLA or shared epitope association in BA, one must consider other possible genetic influences that alter the immune response. Genome-wide association studies have recently been performed to identify disease-specific genetic associations. A Chinese study (Garcia-Barcelo et al. 2010) genotyped nearly half a million single nucleotide polymorphisms (SNPs) in 200 BA patients and 481 controls and identified a strong association of BA with the SNP rs17095355 on chromosome 10q24. Two genes in the region of this SNP include X-prolyl aminopeptidase P1(XPNPEP1) and adducin 3 (ADD3). XPNPEP1 is expressed in biliary epithelia and is involved in the metabolism of inflammatory mediators. Genetic defects of XPNPEP1 could result in deregulation of control of the inflammatory response present in BA. ADD3 is expressed in hepatocytes and biliary epithelia and is involved in the assembly of spectrin-actin membrane protein networks at sites of cell to cell contact. Defects in this gene could theoretically increase fibrosis. Importantly, if there was an HLA association with BA in this Chinese population, it would have been identified within the GWAS analysis detailed above.

Future studies of genetic links to BA that involve alterations of the immune response should include investigations into defects in regulatory T cells (Treg) that would allow inflammation to proceed unchecked. To that end, we have recently identified significant deficits in peripheral blood Treg frequencies in BA infants at the time of diagnosis compared to age-matched controls (Brindley et al. 2012). In the murine model of BA, Lages et al. (2012) showed that adoptive transfer of total CD4^+^ T cells, but not Treg-depleted CD4^+^ T cells, into RRV-infected BA mice was associated with increased survival and decreased bile duct targeted inflammation, suggesting that Tregs protect from bile duct damage. Another avenue of future research into the pathogenesis of BA should include investigations into the possibility that BA is an autoinflammatory disease. Autoinflammatory diseases are not usually HLA-linked and are characterized by exaggerated innate immune responses (Goldbach-Mansky 2012; Rigante 2012).

## Disclaimer

This manuscript was not prepared in collaboration with other ChiLDREN investigators and does not necessarily reflect the opinions or views of ChiLDREN or the NIDDK.

## Electronic supplementary material

Additional file 1: **Frequencies of HLA alleles.** (DOC 197 KB)

Additional file 2: Table S2: HLA allele frequencies of mild versus severe BA. (DOC 248 KB)

## Abbreviations

ADD3: Adducin 3
BA: Biliary Atresia
CHiLDREN: Childhood Liver Disease Research and Education Network
SBT: Sequence based typing
GWAS: Genome-wide association studies
HLA: Human leukocyte antigen
SNPs: Single Nucleotide Polymorphisms
XPNPEP1: X-prolyl aminopeptidase P1.
