# Supplementary material for: Lack of HLA predominance and HLA shared epitopes in biliary Atresia
Source: Springerplus. 2013 Feb 8;2:42. doi: 10.1186/2193-1801-2-42 (PMC3595468; doi:10.1186/2193-1801-2-42)
Supplement: Supplementary file 1 — Additional file 1: Frequencies of HLA alleles. (DOC 197 KB) [file 40064_2012_117_MOESM1_ESM.doc]

|  | **Supplementary Table 1.** **Frequencies of HLA allele A** | | | | | | | | | | | |
| --- | --- | --- | --- | --- | --- | --- | --- | --- | --- | --- | --- | --- |
|  | HLA | | | BA (176)  *n* (%) | Controls (329) *n* (%) | | *Pu* value^#^ | | *Pc* value^#^ | | | |
|  | A | | |  |  | |  | |  | | | |
|  | *01:01 | | | 41(23.3) | 73(22.2) | | 0.78 | | NS | | | |
|  | *02:01 | | | 70(39.8) | 132(40.1) | | 0.94 | | NS | | | |
|  | *02:02 | | | 1(0.6) | 6(1.8) | | 0.43 | | NS | | | |
|  | *02:05 | | | 5(2.8) | 3(0.9) | | 0.13 | | NS | | | |
|  | *02:06 | | | 5(2.8) | 10(3.0) | | 1.00 | | NS | | | |
|  | *02:07 | | | 3(1.7) | 3(0.9) | | 0.42 | | NS | | | |
|  | *03:01 | | | 41(23.3) | 59(17.9) | | 0.15 | | NS | | | |
|  | *03:02 | | | 3(1.7) | 4(1.2) | | 0.70 | | NS | | | |
|  | *11:01 | | | 17(9.7) | 31(9.4) | | 0.93 | | NS | | | |
|  | *23:01 | | | 8(4.5) | 14(4.3) | | 1.00 | | NS | | | |
|  | *24:02 | | | 22(12.5) | 48(14.6) | | 0.52 | | NS | | | |
|  | *25:01 | | | 6(3.4) | 11(3.3) | | 1.00 | | NS | | | |
|  | *26:01 | | | 13(7.4) | 22(6.7) | | 0.77 | | NS | | | |
|  | *29:01 | | | 2(1.1) | 6(1.8) | | 0.72 | | NS | | | |
|  | *29:02 | | | 9(5.1) | 30(9.1) | | 0.11 | | NS | | | |
|  | *30:01 | | | 5(2.8) | 12(3.6) | | 0.80 | | NS | | | |
|  | **30:02* | | | *10(5.7)* | *6(1.8)* | | *0.03* | | *NS* | | | |
|  | *31:01 | | | 5(2.8) | 17(5.2) | | 0.26 | | NS | | | |
|  | *32:01 | | | 8(4.5) | 20(6.1) | | 0.55 | | NS | | | |
|  | *33:01 | | | 6(3.4) | 10(3.0) | | 0.80 | | NS | | | |
|  | *33:03 | | | 9(5.1) | 19(5.8) | | 0.84 | | NS | | | |
|  | *36:01 | | | 2(1.1) | 1(0.3) | | 0.28 | | NS | | | |
|  | *66:01 | | | 5(2.8) | 4(1.2) | | 0.29 | | NS | | | |
|  | *68:01 | | | 9(5.1) | 23(7.0) | | 0.41 | | NS | | | |
|  | *68:02 | | | 7(4.0) | 15(4.6) | | 0.82 | | NS | | | |
|  | *74:01 | | | 0(0.0) | 6(1.8) | | 0.10 | | NS | | | |
|  | # *Pu*, uncorrected *P* values; *Pc*, corrected *P* values | | | | | | | | | | | |
|  | **Frequencies of HLA allele B** | | | | | | | | | | | |
|  | HLA | | BA (176)  *n* (%) | | | Controls (329) *n* (%) | | *Pu* value^#^ | | *Pc* value^#^ | | |
|  | B | |  | | |  | |  | |  | | |
|  | *07:02 | | 37(21.0) | | | 59(17.9) | | 0.40 | | NS | | |
|  | *08:01 | | 29(16.5) | | | 50(15.2) | | 0.71 | | NS | | |
|  | *13:02 | | 8(4.5) | | | 9(2.7) | | 0.31 | | NS | | |
|  | *14:01 | | 7(4.0) | | | 6(1.8) | | 0.15 | | NS | | |
|  | *14:02 | | 14(8.0) | | | 17(5.2) | | 0.21 | | NS | | |
|  | *15:01 | | 16(9.1) | | | 24(7.3) | | 0.48 | | NS | | |
|  | *15:03 | | 3(1.7) | | | 7(2.1) | | 1 | | NS | | |
|  | *15:16 | | 2(1.1) | | | 5(1.5) | | 1 | | NS | | |
|  | *18:01 | | 11(6.3) | | | 19(5.8) | | 0.83 | | NS | | |
|  | *27:05 | | 7(4.0) | | | 17(5.2) | | 0.66 | | NS | | |
|  | *35:01 | | 20(11.4) | | | 57(17.3) | | 0.08 | | NS | | |
|  | *35:02 | | 2(1.1) | | | 5(1.5) | | 1 | | NS | | |
|  | *35:03 | | 5(2.8) | | | 5(1.5) | | 0.33 | | NS | | |
|  | *35:12 | | 3(1.7) | | | 6(1.8) | | 1 | | NS | | |
|  | *37:01 | | 6(3.4) | | | 8(2.4) | | 0.57 | | NS | | |
|  | *38:01 | | 5(2.8) | | | 11(3.3) | | 1 | | NS | | |
|  | *39:01 | | 5(2.8) | | | 13(4.0) | | 0.62 | | NS | | |
|  | *39:06 | | 4(2.3) | | | 4(1.2) | | 0.46 | | NS | | |
|  | *40:01 | | 13(7.4) | | | 23(7.0) | | 0.87 | | NS | | |
|  | *40:02 | | 7(4.0) | | | 12(3.6) | | 0.81 | | NS | | |
|  | *41:02 | | 3(1.7) | | | 5(1.5) | | 1 | | NS | | |
|  | *42:01 | | 5(2.8) | | | 6(1.8) | | 0.53 | | NS | | |
|  | *44:02 | | 23(13.1) | | | 50(15.2) | | 0.52 | | NS | | |
|  | *44:03 | | 19(10.8) | | | 26(7.9) | | 0.28 | | NS | | |
|  | *45:01 | | 2(1.1) | | | 9(2.7) | | 0.34 | | NS | | |
|  | *46:01 | | 4(2.3) | | | 2(0.6) | | 0.19 | | NS | | |
|  | *48:01 | | 1(0.6) | | | 6(1.8) | | 0.43 | | NS | | |
|  | *49:01 | | 6(3.4) | | | 9(2.7) | | 0.78 | | NS | | |
|  | *50:01 | | 4(2.3) | | | 4(1.2) | | 0.46 | | NS | | |
|  | *51:01 | | 7(4.0) | | | 28(8.5) | | 0.06 | | NS | | |
|  | *52:01 | | 5(2.8) | | | 12(3.6) | | 0.80 | | NS | | |
|  | *53:01 | | 6(3.4) | | | 16(4.9) | | 0.50 | | NS | | |
|  | *55:01 | | 5(2.8) | | | 7(2.1) | | 0.76 | | NS | | |
|  | *57:01 | | 4(2.3) | | | 17(5.2) | | 0.16 | | NS | | |
|  | *58:01 | | 8(4.5) | | | 6(1.8) | | 0.09 | | NS | | |
|  | *58:02 | | 3(1.7) | | | 5(1.5) | | 1 | | NS | | |
|  | # *Pu*, uncorrected *P* values; *Pc*, corrected *P* values | | | | | | | | | | | |
| **Frequencies of HLA allele C** | | | | | | | | | | | |  |
| HLA | | BA (177)  *n* (%) | | | | Controls (350) *n* (%) | | *Pu* value^#^ | | | *Pc* value^#^ |  |
| C | |  | | | |  | |  | | |  |  |
| *01:02 | | 11(6.3) | | | | 27(7.7) | | 0.55 | | | NS |  |
| *02:02 | | 11(6.3) | | | | 19(5.4) | | 0.69 | | | NS |  |
| *02:10 | | 1(0.6) | | | | 7(2.0) | | 0.28 | | | NS |  |
| *03:02 | | 6(3.4) | | | | 3(0.9) | | 0.07 | | | NS |  |
| *03:03 | | 20(11.4) | | | | 31(8.9) | | 0.35 | | | NS |  |
| *03:04 | | 20(11.4) | | | | 48(13.7) | | 0.46 | | | NS |  |
| *04:01 | | 39(22.2) | | | | 97(27.7) | | 0.18 | | | NS |  |
| *05:01 | | 25(14.2) | | | | 50(14.3) | | 1 | | | NS |  |
| *06:02 | | 28(15.9) | | | | 53(15.1) | | 0.80 | | | NS |  |
| *07:01 | | 39(22.2) | | | | 67(19.1) | | 0.40 | | | NS |  |
| *07:02 | | 42(23.9) | | | | 77(22.0) | | 0.61 | | | NS |  |
| *07:04 | | 5(2.8) | | | | 13(3.7) | | 0.80 | | | NS |  |
| *07:18 | | 3(1.7) | | | | 7(2.0) | | 1 | | | NS |  |
| *08:01 | | 3(1.7) | | | | 10(2.9) | | 0.56 | | | NS |  |
| *08:02 | | 20(11.4) | | | | 30(8.6) | | 0.29 | | | NS |  |
| *12:02 | | 4(2.3) | | | | 9(2.6) | | 1 | | | NS |  |
| *12:03 | | 13(7.4) | | | | 25(7.1) | | 0.91 | | | NS |  |
| *14:02 | | 5(2.8) | | | | 13(3.7) | | 0.80 | | | NS |  |
| *15:02 | | 6(3.4) | | | | 14(4.0) | | 0.81 | | | NS |  |
| *16:01 | | 15(8.5) | | | | 25(7.1) | | 0.56 | | | NS |  |
| *17:01 | | 7(4.0) | | | | 11(3.1) | | 0.62 | | | NS |  |
| # *Pu*, uncorrected *P* values; *Pc*, corrected *P* values | | | | | | | | | | | |  |
|  | | | | | | | | | | | |  |
| **Frequencies of HLA allele DRB1** | | | | | | | | | | | | |
| HLA | | | BA (178)  *n* (%) | | | Controls (684) *n* (%) | | *Pu* value^#^ | | *Pc* value^#^ | | |
| DRB1 | | |  | | |  | |  | |  | | |
| *01:01 | | | 18(10.1) | | | 85(12.4) | | 0.40 | | NS | | |
| *01:02 | | | 7(3.9) | | | 28(4.1) | | 1 | | NS | | |
| *01:03 | | | 7(3.9) | | | 16(2.3) | | 0.29 | | NS | | |
| *03:01 | | | 34(19.1) | | | 125(18.3) | | 0.80 | | NS | | |
| *03:02 | | | 4(2.2) | | | 14(2.0) | | 0.77 | | NS | | |
| *04:01 | | | 18(10.1) | | | 90(13.2) | | 0.27 | | NS | | |
| *04:02 | | | 2(1.1) | | | 11(1.6) | | 1 | | NS | | |
| *04:03 | | | 5(2.8) | | | 10(1.5) | | 0.21 | | NS | | |
| *04:04 | | | 18(10.1) | | | 47(6.9) | | 0.14 | | NS | | |
| *04:05 | | | 5(2.8) | | | 13(1.9) | | 0.39 | | NS | | |
| *04:07 | | | 4(2.2) | | | 26(3.8) | | 0.49 | | NS | | |
| *07:01 | | | 42(23.6) | | | 152(22.2) | | 0.70 | | NS | | |
| *08:01 | | | 6(3.4) | | | 28(4.1) | | 0.83 | | NS | | |
| *08:02 | | | 4(2.2) | | | 21(3.1) | | 0.80 | | NS | | |
| *08:03 | | | 2(1.1) | | | 13(1.9) | | 0.75 | | NS | | |
| *08:04 | | | 2(1.1) | | | 13(1.9) | | 0.75 | | NS | | |
| *09:01 | | | 7(3.9) | | | 30(4.4) | | 1 | | NS | | |
| *10:01 | | | 4(2.2) | | | 11(1.6) | | 0.53 | | NS | | |
| *11:01 | | | 18(10.1) | | | 73(10.7) | | 0.83 | | NS | | |
| *11:02 | | | 3(1.7) | | | 8(1.2) | | 0.71 | | NS | | |
| *11:04 | | | 12(6.7) | | | 30(4.4) | | 0.24 | | NS | | |
| *12:01 | | | 9(5.1) | | | 29(4.2) | | 0.68 | | NS | | |
| *13:01 | | | 24(13.5) | | | 70(10.2) | | 0.22 | | NS | | |
| *13:02 | | | 12(6.7) | | | 48(7.0) | | 0.90 | | NS | | |
| *13:03 | | | 5(2.8) | | | 20(2.9) | | 1 | | NS | | |
| *14:02 | | | 3(1.7) | | | 7(1.0) | | 0.44 | | NS | | |
| *14:06 | | | 0(0.0) | | | 14(2.0) | | 0.09 | | NS | | |
| *14:54 | | | 5(2.8) | | | 38(5.6) | | 0.18 | | NS | | |
| *15:01 | | | 30(16.9) | | | 131(19.2) | | 0.48 | | NS | | |
| *15:02 | | | 4(2.2) | | | 28(4.1) | | 0.37 | | NS | | |
| **15:03* | | | *11(6.2)* | | | *18(2.6)* | | *0.03* | | *NS* | | |
| *16:01 | | | 5(2.8) | | | 18(2.6) | | 0.80 | | NS | | |
| *16:02 | | | 1(0.6) | | | 13(1.9) | | 0.32 | | NS | | |
| # *Pu*, uncorrected *P* values; *Pc*, corrected *P* values | | | | | | | | | | | | |
| **Frequencies of HLA allele DPB1** | | | | | | | | | | | | |
| HLA | | BA (111)  *n* (%) | | | | Controls (91)  *n* (%) | | *Pu* value^#^ | | *Pc* value^#^ | | |
| DPB1 | |  | | | |  | |  | |  | | |
| *01:01 | | 16(14.4) | | | | 5(5.5) | | 0.06 | | NS | | |
| *02:01 | | 32(28.8) | | | | 21(23.1) | | 0.36 | | NS | | |
| *02:02 | | 1(0.9) | | | | 2(2.2) | | 0.59 | | NS | | |
| *03:01 | | 24(21.6) | | | | 17(18.7) | | 0.61 | | NS | | |
| *04:01 | | 68(61.3) | | | | 62(68.1) | | 0.31 | | NS | | |
| *04:02 | | 23(20.7) | | | | 21(23.1) | | 0.69 | | NS | | |
| *05:01 | | 4(3.6) | | | | 2(2.2) | | 0.69 | | NS | | |
| *06:01 | | 3(2.7) | | | | 1(1.1) | | 0.63 | | NS | | |
| *09:01 | | 1(0.9) | | | | 3(3.3) | | 0.33 | | NS | | |
| *10:01 | | 1(0.9) | | | | 6(6.6) | | 0.05 | | NS | | |
| *11:01 | | 6(5.4) | | | | 2(2.2) | | 0.30 | | NS | | |
| *13:01 | | 2(1.8) | | | | 3(3.3) | | 0.66 | | NS | | |
| *14:01 | | 3(2.7) | | | | 1(1.1) | | 0.63 | | NS | | |
| *15:01 | | 4(3.6) | | | | 0(0) | | 0.13 | | NS | | |
| *16:01 | | 3(2.7) | | | | 0(0) | | 0.25 | | NS | | |
| *17:01 | | 3(2.7) | | | | 1(1.1) | | 0.63 | | NS | | |
| *20:01 | | 1(0.9) | | | | 3(3.3) | | 0.33 | | NS | | |
| *104:01 | | 1(0.9) | | | | 2(2.2) | | 0.59 | | NS | | |
| # *Pu*, uncorrected *P* values; *Pc*, corrected *P* values | | | | | | | | | | | | |
| **Frequencies of HLA allele DQB1** | | | | | | | | | | | | |
| HLA | | | BA (178)  *n* (%) | | | Controls (342) *n* (%) | | *Pu* value^#^ | | *Pc* value^#^ | | |
| DQB1 | | |  | | |  | |  | |  | | |
| *02:01 | | | 34(19.1) | | | 62(18.1) | | 0.83 | | NS | | |
| *02:02 | | | 37(20.8) | | | 68(19.9) | | 0.85 | | NS | | |
| *03:01 | | | 57(32.0) | | | 108(31.6) | | 0.98 | | NS | | |
| *03:02 | | | 34(19.1) | | | 65(19.0) | | 0.97 | | NS | | |
| *03:03 | | | 11(6.2) | | | 28(8.2) | | 0.39 | | NS | | |
| *03:19 | | | 4(2.2) | | | 12(3.5) | | 0.60 | | NS | | |
| *04:02 | | | 13(7.3) | | | 30(8.8) | | 0.54 | | NS | | |
| *05:01 | | | 40(22.5) | | | 85(24.9) | | 0.49 | | NS | | |
| *05:02 | | | 12(6.7) | | | 18(5.3) | | 0.51 | | NS | | |
| *05:03 | | | 8(4.5) | | | 25(7.3) | | 0.20 | | NS | | |
| *06:01 | | | 5(2.8) | | | 16(4.7) | | 0.36 | | NS | | |
| *06:02 | | | 45(25.3) | | | 69(20.2) | | 0.19 | | NS | | |
| *06:03 | | | 21(11.8) | | | 32(9.4) | | 0.40 | | NS | | |
| *06:04 | | | 10(5.6) | | | 9(2.6) | | 0.14 | | NS | | |
| *06:09 | | | 3(1.7) | | | 7(2.0) | | 1 | | NS | | |
| # *Pu*, uncorrected *P* values; *Pc*, corrected *P* values | | | | | | | | | | | | |
