# Supplementary material for: Lack of HLA predominance and HLA shared epitopes in biliary Atresia
Source: Springerplus. 2013 Feb 8;2:42. doi: 10.1186/2193-1801-2-42 (PMC3595468; doi:10.1186/2193-1801-2-42)
Supplement: Supplementary file 2 — Additional file 2: Table S2: HLA allele frequencies of mild versus severe BA. (DOC 248 KB) [file 40064_2012_117_MOESM2_ESM.doc]

**Supplementary Table 2. HLA allele frequencies of mild versus severe BA**

| HLA | Mild BA (70)  n (%) | | Severe BA (78)  n (%) | | | *Pu* value* | | | *Pc* value* |
| --- | --- | --- | --- | --- | --- | --- | --- | --- | --- |
| A |  | |  | | |  | | |  |
| *01:01 | 17(24.29) | | 17(21.79) | | | 0.72 | | | NS |
| *01:02 | 0(0) | | 1(1.28) | | | 1.00 | | | NS |
| *02:01 | 30(42.86) | | 25(32.05) | | | 0.17 | | | NS |
| *02:03 | 1(1.43) | | 0(0) | | | 0.47 | | | NS |
| *02:05 | 2(2.86) | | 2(2.56) | | | 1.00 | | | NS |
| *02:06 | 2(2.86) | | 2(2.56) | | | 1.00 | | | NS |
| *02:07 | 1(1.43) | | 2(2.56) | | | 1.00 | | | NS |
| *02:08 | 1(1.43) | | 0(0) | | | 0.47 | | | NS |
| *03:01 | 21(30.00) | | 15(19.23) | | | 0.13 | | | NS |
| *03:02 | 1(1.43) | | 0(0) | | | 0.47 | | | NS |
| *11:01 | 6(8.57) | | 9(11.54) | | | 0.60 | | | NS |
| *11:02 | 1(1.43) | | 0(0) | | | 0.47 | | | NS |
| *23:01 | 2(2.86) | | 6(7.69) | | | 0.28 | | | NS |
| *23:17 | 0(0) | | 1(1.28) | | | 1.00 | | | NS |
| *24:02 | 5(7.14) | | 14(17.95) | | | 0.08 | | | NS |
| *24:07 | 1(1.43) | | 0(0) | | | 0.47 | | | NS |
| *25:01 | 2(2.86) | | 2(2.56) | | | 1.00 | | | NS |
| *26:01 | 7(10.00) | | 4(5.13) | | | 0.35 | | | NS |
| *29:01 | 1(1.43) | | 1(1.28) | | | 1.00 | | | NS |
| *29:02 | 4(5.71) | | 4(5.13) | | | 1.00 | | | NS |
| *30:01 | 2(2.86) | | 2(2.56) | | | 1.00 | | | NS |
| *30:02 | 2(2.86) | | 7(8.97) | | | 0.17 | | | NS |
| *30:04 | 0(0) | | 1(1.28) | | | 1.00 | | | NS |
| *31:01 | 1(1.43) | | 3(3.85) | | | 0.62 | | | NS |
| *32:01 | 2(2.86) | | 6(7.69) | | | 0.28 | | | NS |
| *33:01 | 2(2.86) | | 4(5.13) | | | 0.68 | | | NS |
| *33:03 | 5(7.14) | | 2(2.56) | | | 0.26 | | | NS |
| *34:02 | 0(0) | | 2(2.56) | | | 0.50 | | | NS |
| *36:01 | 0(0) | | 1(1.28) | | | 1.00 | | | NS |
| *66:01 | 1(1.43) | | 2(2.56) | | | 1.00 | | | NS |
| *68:01 | 3(4.29) | | 6(7.69) | | | 0.50 | | | NS |
| *68:02 | 3(4.29) | | 3(3.85) | | | 1.00 | | | NS |
| *68:03 | 0(0) | | 1(1.28) | | | 1.00 | | | NS |
| *80:01 | 1(1.43) | | 0(0) | | | 0.47 | | | NS |
| **Pu*, uncorrected *P* values; *Pc*, corrected *P* values | | | | | | | | | |
| HLA | | Mild BA (70)  n (%) | | | Severe BA (78)  n (%) | | *Pu* value* | | *Pc* value* |
| B | |  | | |  | |  | |  |
| *07:02 | | 15(21.43) | | | 16(20.51) | | 0.89 | | NS |
| *07:07 | | 1(1.43) | | | 0(0) | | 0.47 | | NS |
| *08:01 | | 12(17.14) | | | 11(14.10) | | 0.61 | | NS |
| *13:01 | | 0(0) | | | 1(1.28) | | 1.00 | | NS |
| *13:02 | | 3(4.29) | | | 4(5.13) | | 1.00 | | NS |
| *14:01 | | 1(1.43) | | | 6(7.69) | | 0.12 | | NS |
| *14:02 | | 8(11.43) | | | 6(7.69) | | 0.58 | | NS |
| *15:01 | | 5(7.14) | | | 10(12.82) | | 0.29 | | NS |
| *15:03 | | 1(1.43) | | | 0(0) | | 0.47 | | NS |
| *15:10 | | 2(2.86) | | | 1(1.28) | | 0.60 | | NS |
| *15:16 | | 1(1.43) | | | 1(1.28) | | 1.00 | | NS |
| *15:17 | | 1(1.43) | | | 0(0) | | 0.47 | | NS |
| *15:18 | | 0(0) | | | 1(1.28) | | 1.00 | | NS |
| *15:24 | | 0(0) | | | 2(2.56) | | 0.50 | | NS |
| *15:30 | | 1(1.43) | | | 0(0) | | 0.47 | | NS |
| *15:32 | | 1(1.43) | | | 0(0) | | 0.47 | | NS |
| *18:01 | | 3(4.29) | | | 5(6.41) | | 0.72 | | NS |
| *27:04 | | 1(1.43) | | | 0(0) | | 0.47 | | NS |
| *27:05 | | 3(4.29) | | | 4(5.13) | | 1.00 | | NS |
| *35:01 | | 7(10.00) | | | 10(12.82) | | 0.62 | | NS |
| *35:02 | | 0(0) | | | 1(1.28) | | 1.00 | | NS |
| *35:03 | | 1(1.43) | | | 3(3.85) | | 0.62 | | NS |
| *35:05 | | 1(1.43) | | | 0(0) | | 0.47 | | NS |
| *35:12 | | 1(1.43) | | | 1(1.28) | | 1.00 | | NS |
| *37:01 | | 2(2.86) | | | 2(2.56) | | 1.00 | | NS |
| *38:01 | | 1(1.43) | | | 2(2.56) | | 1.00 | | NS |
| *38:02 | | 1(1.43) | | | 0(0) | | 0.47 | | NS |
| *39:01 | | 4(5.71) | | | 1(1.28) | | 0.19 | | NS |
| *39:06 | | 0(0) | | | 4(5.13) | | 0.12 | | NS |
| *39:10 | | 0(0) | | | 1(1.28) | | 1.00 | | NS |
| *40:01 | | 7(10.00) | | | 4(5.13) | | 0.35 | | NS |
| *40:02 | | 0(0) | | | 6(7.69) | | 0.03 | | NS |
| *40:06 | | 1(1.43) | | | 0(0) | | 0.47 | | NS |
| *40:10 | | 0(0) | | | 1(1.28) | | 1.00 | | NS |
| *41:02 | | 2(2.86) | | | 1(1.28) | | 0.60 | | NS |
| *42:01 | | 4(5.71) | | | 1(1.28) | | 0.19 | | NS |
| *44:02 | | 11(15.71) | | | 6(7.69) | | 0.20 | | NS |
| *44:03 | | 7(10.00) | | | 9(11.54) | | 0.80 | | NS |
| *44:05 | | 1(1.43) | | | 0(0) | | 0.47 | | NS |
| *45:01 | | 0(0) | | | 2(2.56) | | 0.50 | | NS |
| *46:01 | | 2(2.86) | | | 2(2.56) | | 1.00 | | NS |
| *48:01 | | 0(0) | | | 1(1.28) | | 1.00 | | NS |
| *49:01 | | 1(1.43) | | | 4(5.13) | | 0.37 | | NS |
| *50:01 | | 2(2.86) | | | 2(2.56) | | 1.00 | | NS |
| *51:01 | | 7(10.00) | | | 0(0) | | 0.00 | | NS |
| *51:02 | | 0(0) | | | 1(1.28) | | 1.00 | | NS |
| *51:09 | | 0(0) | | | 1(1.28) | | 1.00 | | NS |
| *52:01 | | 2(2.86) | | | 2(2.56) | | 1.00 | | NS |
| *53:01 | | 0(0) | | | 4(5.13) | | 0.12 | | NS |
| *54:01 | | 1(1.43) | | | 0(0) | | 0.47 | | NS |
| *55:01 | | 2(2.86) | | | 2(2.56) | | 1.00 | | NS |
| *56:01 | | 0(0) | | | 1(1.28) | | 1.00 | | NS |
| *57:01 | | 2(2.86) | | | 2(2.56) | | 1.00 | | NS |
| *57:03 | | 1(1.43) | | | 1(1.28) | | 1.00 | | NS |
| *57:04 | | 0(0) | | | 1(1.28) | | 1.00 | | NS |
| *58:01 | | 4(5.71) | | | 4(5.13) | | 1.00 | | NS |
| *58:02 | | 0(0) | | | 2(2.56) | | 0.50 | | NS |
| *73:01 | | 0(0) | | | 1(1.28) | | 1.00 | | NS |
| *81:01 | | 1(1.43) | | | 0(0) | | 0.47 | | NS |
| **Pu*, uncorrected *P* values; *Pc*, corrected *P* values | | | | | | | | | |
| HLA | | Mild BA (70)  n (%) | | | Severe BA (78)  n (%) | | *Pu* value* | | *Pc* value* |
| C | |  | | |  | |  | |  |
| *01:02 | | 7(10.00) | | | 2(2.56) | | 0.08 | | NS |
| *01:03 | | 0(0) | | | 1(1.28) | | 1.00 | | NS |
| *02:02 | | 4(5.71) | | | 5(6.41) | | 1.00 | | NS |
| *02:10 | | 1(1.43) | | | 0(0) | | 0.47 | | NS |
| *03:02 | | 3(4.29) | | | 3(3.85) | | 1.00 | | NS |
| *03:03 | | 6(8.57) | | | 10(12.82) | | 0.44 | | NS |
| *03:04 | | 9(12.86) | | | 9(11.54) | | 0.81 | | NS |
| *03:07 | | 0(0) | | | 1(1.28) | | 1.00 | | NS |
| *04:01 | | 12(17.14) | | | 18(23.08) | | 0.37 | | NS |
| *04:03 | | 0(0) | | | 1(1.28) | | 1.00 | | NS |
| *05:01 | | 10(14.29) | | | 8(10.26) | | 0.46 | | NS |
| *06:02 | | 8(11.43) | | | 16(20.51) | | 0.13 | | NS |
| *07:01 | | 15(21.43) | | | 18(23.08) | | 0.81 | | NS |
| *07:02 | | 18(25.71) | | | 19(24.36) | | 0.85 | | NS |
| *07:04 | | 1(1.43) | | | 3(3.85) | | 0.62 | | NS |
| *07:06 | | 1(1.43) | | | 0(0) | | 0.47 | | NS |
| *07:18 | | 2(2.86) | | | 1(1.28) | | 0.60 | | NS |
| *08:01 | | 1(1.43) | | | 2(2.56) | | 1.00 | | NS |
| *08:02 | | 9(12.86) | | | 11(14.10) | | 1.00 | | NS |
| *08:04 | | 1(1.43) | | | 0(0) | | 0.47 | | NS |
| *12:02 | | 3(4.29) | | | 1(1.28) | | 0.34 | | NS |
| *12:03 | | 5(7.14) | | | 4(5.13) | | 0.74 | | NS |
| *14:02 | | 5(7.14) | | | 0(0) | | 0.02 | | NS |
| *15:02 | | 3(4.29) | | | 2(2.56) | | 0.67 | | NS |
| *15:05 | | 0(0) | | | 1(1.28) | | 1.00 | | NS |
| *16:01 | | 5(7.14) | | | 8(10.26) | | 0.57 | | NS |
| *16:02 | | 0(0) | | | 1(1.28) | | 1.00 | | NS |
| *17:01 | | 4(5.71) | | | 2(2.56) | | 0.42 | | NS |
| *17:03 | | 2(2.86) | | | 0(0) | | 0.22 | | NS |
| *18:01 | | 1(1.43) | | | 0(0) | | 0.47 | | NS |
| *18:02 | | 0(0) | | | 2(2.56) | | 0.50 | | NS |
| **Pu*, uncorrected *P* values; *Pc*, corrected *P* values | | | | | | | | | |
| HLA | | Mild BA (70)  n (%) | | | Severe BA (78)  n (%) | | | *Pu* value* | *Pc* value* |
| DRB1 | |  | | |  | | |  |  |
| *01:01 | | 8(11.43) | | | 9(11.54) | | | 1.00 | NS |
| *01:02 | | 4(5.71) | | | 3(3.85) | | | 0.71 | NS |
| *01:03 | | 2(2.86) | | | 5(6.41) | | | 0.45 | NS |
| *03:01 | | 14(20.00) | | | 12(15.38) | | | 0.46 | NS |
| *03:02 | | 3(4.29) | | | 1(1.28) | | | 0.34 | NS |
| *04:01 | | 7(10.00) | | | 7(8.97) | | | 1.00 | NS |
| *04:02 | | 2(2.86) | | | 0(0) | | | 0.22 | NS |
| *04:03 | | 4(5.71) | | | 1(1.28) | | | 0.19 | NS |
| *04:04 | | 4(5.71) | | | 9(11.54) | | | 0.25 | NS |
| *04:05 | | 2(2.86) | | | 3(3.85) | | | 1.00 | NS |
| *04:06 | | 2(2.86) | | | 0(0) | | | 0.22 | NS |
| *04:07 | | 2(2.86) | | | 1(1.28) | | | 0.60 | NS |
| *04:08 | | 2(2.86) | | | 0(0) | | | 0.22 | NS |
| *04:11 | | 0(0) | | | 1(1.28) | | | 1.00 | NS |
| *07:01 | | 13(18.57) | | | 13(29.49) | | | 0.12 | NS |
| *08:01 | | 4(5.71) | | | 1(1.28) | | | 0.19 | NS |
| *08:02 | | 1(1.43) | | | 2(2.56) | | | 1.00 | NS |
| *08:03 | | 1(1.43) | | | 1(1.28) | | | 1.00 | NS |
| *08:04 | | 1(1.43) | | | 1(1.28) | | | 1.00 | NS |
| *08:10 | | 1(1.43) | | | 0(0) | | | 0.47 | NS |
| *09:01 | | 3(4.29) | | | 4(5.13) | | | 1.00 | NS |
| *10:01 | | 1(1.43) | | | 2(2.56) | | | 1.00 | NS |
| *11:01 | | 7(10.00) | | | 8(10.26) | | | 1.00 | NS |
| *11:02 | | 0(0) | | | 3(3.85) | | | 0.25 | NS |
| *11:03 | | 1(1.43) | | | 0(0) | | | 0.47 | NS |
| *11:04 | | 6(8.57) | | | 4(5.13) | | | 0.52 | NS |
| *12:01 | | 3(4.29) | | | 5(6.41) | | | 0.72 | NS |
| *13:01 | | 5(7.14) | | | 12(15.38) | | | 0.13 | NS |
| *13:02 | | 6(8.57) | | | 4(5.13) | | | 0.52 | NS |
| *13:03 | | 3(4.29) | | | 2(2.56) | | | 0.67 | NS |
| *14:01 | | 0(0) | | | 2(2.56) | | | 0.50 | NS |
| *14:02 | | 0(0) | | | 2(2.56) | | | 0.50 | NS |
| *14:04 | | 0(0) | | | 1(1.28) | | | 1.00 | NS |
| *14:05 | | 1(1.43) | | | 0(0) | | | 0.47 | NS |
| *14:54 | | 2(2.86) | | | 2(2.56) | | | 1.00 | NS |
| *15:01 | | 13(18.57) | | | 9(11.54) | | | 0.23 | NS |
| *15:02 | | 2(2.86) | | | 2(2.56) | | | 1.00 | NS |
| *15:03 | | 4(5.71) | | | 5(6.41) | | | 1.00 | NS |
| *16:01 | | 3(4.29) | | | 1(1.28) | | | 0.34 | NS |
| *16:02 | | 0(0) | | | 1(1.28) | | | 1.00 | NS |
| **Pu*, uncorrected *P* values; *Pc*, corrected *P* values | | | | | | | | | |
| HLA | | Mild BA (70)  n (%) | | Severe BA (78)  n (%) | | | *Pu* value* | | *Pc* value* |
| DPB1 | |  | |  | | |  | |  |
| *01:01 | | 12(17.14) | | 17(20.51) | | | 0.57 | | NS |
| *02:01 | | 20(28.57) | | 22(26.92) | | | 0.86 | | NS |
| *02:02 | | 2(2.86) | | 3(2.56) | | | 1.00 | | NS |
| *03:01 | | 4(20.00) | | 14(16.67) | | | 0.63 | | NS |
| *04:01 | | 36(51.43) | | 36(44.87) | | | 0.47 | | NS |
| *04:02 | | 17(24.29) | | 18(21.79) | | | 0.75 | | NS |
| *05:01 | | 3(4.29) | | 6(6.41) | | | 0.72 | | NS |
| *06:01 | | 1(1.43) | | 2(1.28) | | | 1.00 | | NS |
| *09:01 | | 1(1.43) | | 3(2.56) | | | 1.00 | | NS |
| *10:01 | | 0(0) | | 2(1.28) | | | 1.00 | | NS |
| 104:01 | | 1(1.43) | | 2(1.28) | | | 1.00 | | NS |
| *11:01 | | 5(7.14) | | 3(2.56) | | | 0.26 | | NS |
| *13:01 | | 4(5.71) | | 5(5.13) | | | 1.00 | | NS |
| *14:01 | | 3(4.29) | | 2(1.28) | | | 0.35 | | NS |
| *15:01 | | 2(2.86) | | 3(2.56) | | | 1.00 | | NS |
| *16:01 | | 1(1.43) | | 3(2.56) | | | 1.00 | | NS |
| *17:01 | | 3(4.29) | | 4(3.85) | | | 1.00 | | NS |
| *18:01 | | 0(0) | | 6(6.41) | | | 0.06 | | NS |
| *19:01 | | 1(1.43) | | 1(0) | | | 0.48 | | NS |
| *20:01 | | 0(0) | | 2(1.28) | | | 1.00 | | NS |
| *23:01 | | 0(0) | | 2(1.28) | | | 1.00 | | NS |
| *39:01 | | 1(1.43) | | 1(0) | | | 0.48 | | NS |
| *85:01 | | 0(0) | | 2(1.28) | | | 1.00 | | NS |
| **Pu*, uncorrected *P* values; *Pc*, corrected *P* values | | | | | | | | | |
| HLA | | Mild BA (70)  n (%) | | Severe BA (78)  n (%) | | | *Pu* value* | | *Pc* value* |
| DQB1 | |  | |  | | |  | |  |
| *02:01 | | 14(20.00) | | 12(15.38) | | | 0.46 | | NS |
| *02:02 | | 11(15.71) | | 21(26.92) | | | 0.10 | | NS |
| *03:01 | | 27(38.57) | | 23(29.49) | | | 0.24 | | NS |
| *03:02 | | 13(18.57) | | 14(17.95) | | | 0.92 | | NS |
| *03:03 | | 5(7.14) | | 5(6.41) | | | 1.00 | | NS |
| *03:19 | | 0(0) | | 4(5.13) | | | 0.12 | | NS |
| *04:02 | | 7(10.00) | | 4(5.13) | | | 0.35 | | NS |
| *05:01 | | 16(22.86) | | 21(26.92) | | | 0.57 | | NS |
| *05:02 | | 7(10.00) | | 4(5.13) | | | 0.35 | | NS |
| *05:03 | | 2(2.86) | | 4(5.13) | | | 0.68 | | NS |
| *06:01 | | 3(4.29) | | 2(2.56) | | | 0.67 | | NS |
| *06:02 | | 15(21.43) | | 17(21.79) | | | 0.96 | | NS |
| *06:03 | | 5(7.14) | | 10(12.82) | | | 0.29 | | NS |
| *06:04 | | 6(8.57) | | 3(3.85) | | | 0.31 | | NS |
| *06:08 | | 0(0) | | 1(1.28) | | | 1.00 | | NS |
| *06:09 | | 0(0) | | 1(1.28) | | | 1.00 | | NS |
| **Pu*, uncorrected *P* values; *Pc*, corrected *P* values | | | | | | | | | |
